# *Bartonella* spp. in Fruit Bats and Blood-Feeding Ectoparasites in Madagascar

**DOI:** 10.1371/journal.pntd.0003532

**Published:** 2015-02-23

**Authors:** Cara E. Brook, Ying Bai, Andrew P. Dobson, Lynn M. Osikowicz, Hafaliana C. Ranaivoson, Qiyun Zhu, Michael Y. Kosoy, Katharina Dittmar

**Affiliations:** 1 Department of Ecology and Evolutionary Biology, Princeton University, Princeton, New Jersey, United States of America; 2 Division of Vector-Borne Diseases, Centers for Disease Control and Prevention, Fort Collins, Colorado, United States of America; 3 Virology Unit, Institut Pasteur de Madagascar, Antananarivo, Madagascar; 4 Department of Biological Sciences, University at Buffalo, Buffalo, New York, United States of America; University of California San Diego School of Medicine, UNITED STATES

## Abstract

We captured, ectoparasite-combed, and blood-sampled cave-roosting Madagascan fruit bats (*Eidolon dupreanum*) and tree-roosting Madagascan flying foxes (*Pteropus rufus*) in four single-species roosts within a sympatric geographic foraging range for these species in central Madagascar. We describe infection with novel *Bartonella* spp. in sampled *Eidolon dupreanum* and associated bat flies (*Cyclopodia dubia*), which nest close to or within major known *Bartonella* lineages; simultaneously, we report the absence of *Bartonella* spp. in *Thaumapsylla* sp. fleas collected from these same bats. This represents the first documented finding of *Bartonella* infection in these species of bat and bat fly, as well as a new geographic record for *Thaumapsylla* sp. We further relate the absence of both *Bartonella* spp. and ectoparasites in sympatrically sampled *Pteropus rufus*, thus suggestive of a potential role for bat flies in *Bartonella* spp. transmission. These findings shed light on transmission ecology of bat-borne *Bartonella* spp., recently demonstrated as a potentially zoonotic pathogen.

## Author Summary

*Bartonella* spp. are bacteria that inhabit the red blood cells of both human and animal hosts. Among humans, *Bartonella* spp. are known to cause several febrile illnesses, including Carrion’s disease (*Bartonella bacilliformis*), trench fever (*Bartonella quintana*), and cat scratch fever (*Bartonella henselae*), all of which are transmitted via arthropod vectors—respectively sand flies, lice, and fleas. Bats are known to host multiple *Bartonella* spp., including some capable of infecting humans. Some bat species are also known to host obligate ectoparasites known as bat flies (Diptera: Hippoboscoidea), which also sometimes support *Bartonella* spp. infections. The role of bat flies and other bat ectoparasites as vectors for *Bartonella* spp. transmission has been suggested, but not fully explored. We demonstrate *Bartonella* spp. infection in one species of Madagascar fruit bat, which hosts bat flies, simultaneously with the absence of *Bartonella* in a fruit bat species of overlapping range that appears not to support these ectoparasites. In light of ongoing trends of zoonotic emergence of human diseases from bat reservoirs, further understanding of the transmission dynamics of bat-borne pathogens is paramount.

## Introduction

The role of bats as reservoirs for viral pathogens—including several responsible for severe human disease—has received increasing attention in recent years [1]. The extent to which this pattern is mirrored by bats’ abilities to host and transmit other zoonotic agents, including bacteria, is less widely acknowledged. Bats have been confirmed as asymptomatic reservoirs for several species of gram-negative *Bartonella* bacteria in localities as wide-ranging as the United Kingdom [2], Kenya [3], Guatemala [4], Peru [5], Taiwan [6], Nigeria [7], and Puerto Rico [8]. *Bartonella* spp. infect erythrocytes and epithelial cells of predominantly mammalian hosts, and some are known to cause zoonotic disease (bartonellosis) in humans. Most recently, bats in the Northern Hemisphere have been implicated as hosts for the human pathogen, *Bartonella mayotimonensis*, although the mechanism of transmission between bats and humans remains unclear [9]. *Bartonella* spp. are frequently transmitted via arthropod vectors [10] and have been identified in several bat ectoparasites, including 19 bat fly species (Diptera: Hippoboscoidea: Nycteribiidae and Streblidae) [11,12]. However, the presence of *Bartonella* spp. within these arthropods may simply reflect their ingestion of host blood, and vector transmission of *Bartonella* between bats has yet to be confirmed via experimental trial or controlled field study. Nonetheless, phylogenetic analyses of global bat fly-*Bartonella*-bat associations demonstrate *Bartonella* spp. similarities across bat hosts and ectoparasites [12], suggesting that bat flies might play a vector role in transmission. To elucidate this relationship, we examined *Bartonella* prevalence in two sympatric Madagascar fruit bat species—one containing bat flies and fleas and one in which ectoparasites were conspicuously absent. Here we report the presence of closely related *Bartonella* genotypes in Madagascan fruit bats (*Eidolon dupreanum*) and their associated bat flies (Nycteribiidae) in Madagascar. We simultaneously report the absence of *Bartonella* spp. in bat fleas (*Thaumapsylla* sp.) of *E*. *dupreanum*, in addition to the concomitant absence of both ectoparasites and *Bartonella* in sympatric Madagascan flying foxes (*Pteropus rufus*).

## Materials and Methods

### Bat Capture and Sampling

**Fig 1:**
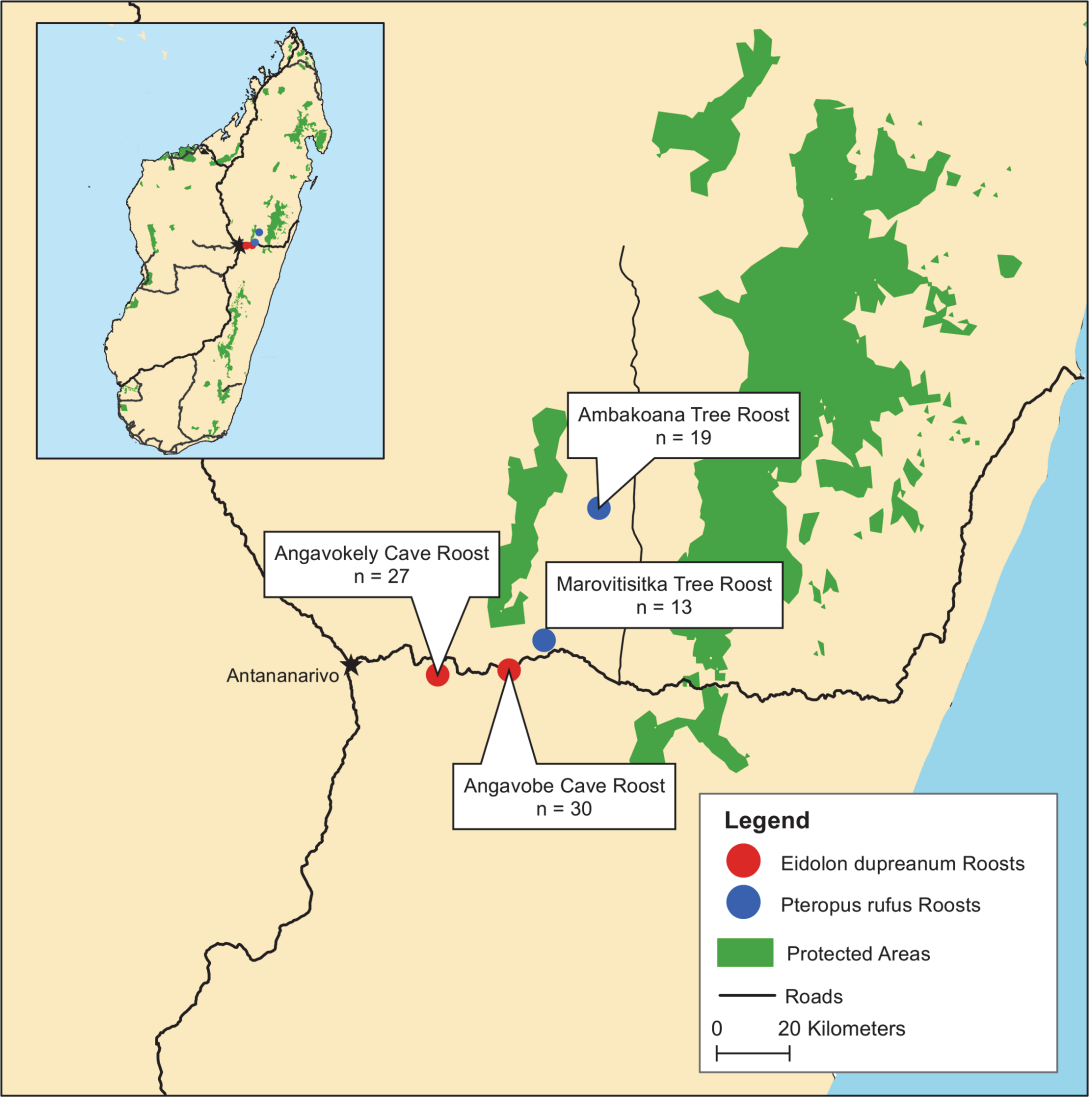
Sites of bat collection, showing numbers of bats collected. Madagascar, 2013.

In November 2013, 57 *E*. *dupreanum* and 32 *P*. *rufus* were mist-netted, sampled for pathogens, and live-released from four single-species roost sites in central Madagascar. *E*. *dupreanum* bats were captured from two cave roosts (Angavobe -18.918050S, 47.94360E; and Angavokely 18.932450 S, 47.7574170 E) and *P*. *rufus* bats netted from two tree roosts (Marovitsika -18.842180S, 48.033630E; and Ambakoana -18.511280S; 48.171120E) in the District of Moramanga. All four roost sites are within a 35km radius of one another and a 5km radius of neighboring human communities, distances well within the nightly foraging ranges of these flying foxes (Fig. 1) [13]. This highland region is dominated by savannah grassland interspersed with non-native plantation and mid-elevation (~1100m) humid forest. Both *E*. *dupreanum* and *P*. *rufus* feed on a range of fruits and nectars and are known to share feeding sites [13].

Upon capture, bats were thoroughly examined for ectoparasites, and all observed flies, fleas, and mites were removed and collected into vials of absolute ethanol with a comb (fleas) or tweezers (mites and bat flies). Blood (1.0ml) was collected from the brachial vein of adult bats (forearm >100mm) and robust juveniles (29 *P*. *rufus*, 47 *E*. *dupreanum* blood-sampled). Serum and blood cells were separated by centrifuging and stored in liquid nitrogen in the field, then transferred to -80°C freezers at the Institut Pasteur-Madagascar.

### Ethics Statement

This study was carried out in strict accordance with guidelines posted by the American Veterinary Medical Association. All field protocols employed were pre-approved by the Princeton University Institutional Animal Care and Use Committee (IACUC Protocol # 1926), and every effort was made to minimize discomfort to animals.

### Sample Processing and Molecular Analysis

**Bat flies**. Ectoparasite samples were processed at the University at Buffalo (Buffalo, NY, USA). Ectoparasite DNA was extracted from a subset of samples (19 bat flies and 6 fleas) using the Qiagen Animal Tissue kit (QIAGEN, Valencia, CA, USA). Ectoparasite voucher specimens were slide-mounted and identified using available taxonomic keys.

**Blood pellets**. Blood pellet samples were processed at the CDC’s Division of Vector-Borne Diseases (Fort Collins, CO, USA). DNA was extracted from blood samples using a Qiagen QIAamp tissue kit (QIAGEN, Valencia, CA, USA) according to the manufacturer’s instructions.

***Bartonella* spp. assay**. All DNA extractions (ectoparasites and blood) were examined for *Bartonella* spp. by conventional PCR targeting multiple genes employed in previous research: *gltA*, *ftsZ*, and *nuoG* genes for arthropod bartonellae, and *gltA* and ITS sequence for blood samples [3,11,12]. Only samples with sequences that unequivocally BLASTed to *Bartonella* spp. and nested within known *Bartonella* sequences by phylogenetic analysis were considered positive (RAxML 7.7) [14]. Samples positive by PCR with inconclusive sequence data were thus considered negative for *Bartonella* spp. in our analysis.

### Statistical Analysis

We compared the frequency of bat fly (*C*. *dubia*) and bat flea (*Thaumapsylla* sp.) infections, as well as *Bartonella* spp. prevalence in both bat hosts and in ectoparasite arthropods. Differences were examined between species and across sampling sites using chi-squared and Fisher exact tests in the statistical program R [15]. We used a p-value threshold of 0.01 to assess whether observed ectoparasite burden and *Bartonella* spp. prevalence were independent of species and sampling site.

## Results

Seven of 24 (29.2%) *Eidolon dupreanum* sampled from Angavobe cave and 20 of 23 (87%) *E*. *dupreanum* sampled from Angavokely cave were found to host *Cyclopodia dubia* (Nycteribiidae) bat flies. Ten of those 23 (43.5.1%) Angavokely *E*. *dupreanum* also hosted *Thaumapsylla* sp. fleas (Table 1). Both frequency of bat fly and flea hosting varied significantly by roosting site, via analysis by chi-squared tests of independence (bat fly: X^2^ = 13.768, df = 1, p = 0.0002; flea: X^2^ = 10.7863, df = 1, p = 0.001) and Fisher’s exact tests (bat fly: p = 8.828e-05; flea: p = 0.0002). Two of 2 (100%) bat flies processed from Angavobe and 15 of 17 (88.2%) bat flies processed from Angavokely were considered positive for *Bartonella* DNA by sequence, although all bat flies processed were *Bartonella* spp. positive by PCR alone. None of the six *Thaumapsylla* fleas processed were positive for any *Bartonella* target gene. The presence of *Thaumapsylla* sp. at the Angavokely site represents the first geographic record for Madagascar; this genus is known from *Eidolon* spp. elsewhere [16].

**Table 1:** Ectoparasites and *Bartonella* spp. in Madagascar fruit bats.

		Ectoparasite presence		*Bartonella* spp. prevalence		
Bat species	Locality	*C. dubia*	*Thaumapsylla* sp.	In bat host	In *C*. *dubia*	In *Thaumapsylla* sp.
*E. dupreanum*	Angavobe	7/24 (29.2)*	0/24 (0)^*†*^	8/24 (33.3)	2/2 (100)	—
	Angavokely	20/23 (87.0)*	10/23 (43.5) ^*†*^	13/23 (56.5)	15/17 (88.2)	0/6 (0)
*P. rufus*	Marovitsika	0 (0)	0 (0)	0/12 (0)	—	—
	Ambakoana	0 (0)	0 (0)	0/17 (0)	—	—

*Between site differences statistically significant via chi-squared test for independence (X^2^ = 13.768, df = 1, p = 0.0002;) and Fisher’s exact test (p = 8.828e-05)

^*†*^Between site differences statistically significant via chi-squared test for independence (X^2^ = 10.7863, df = 1, p-value = 0.001) and Fisher’s exact test (p = 0.0002)

Table data indicate number positive/number sampled (%) for ectoparasite presence and *Bartonella* spp. prevalence (both in bat host and in hosted ectoparasites) for *E*. *dupreanum* and *P*. *rufus*.

**Fig 2:**
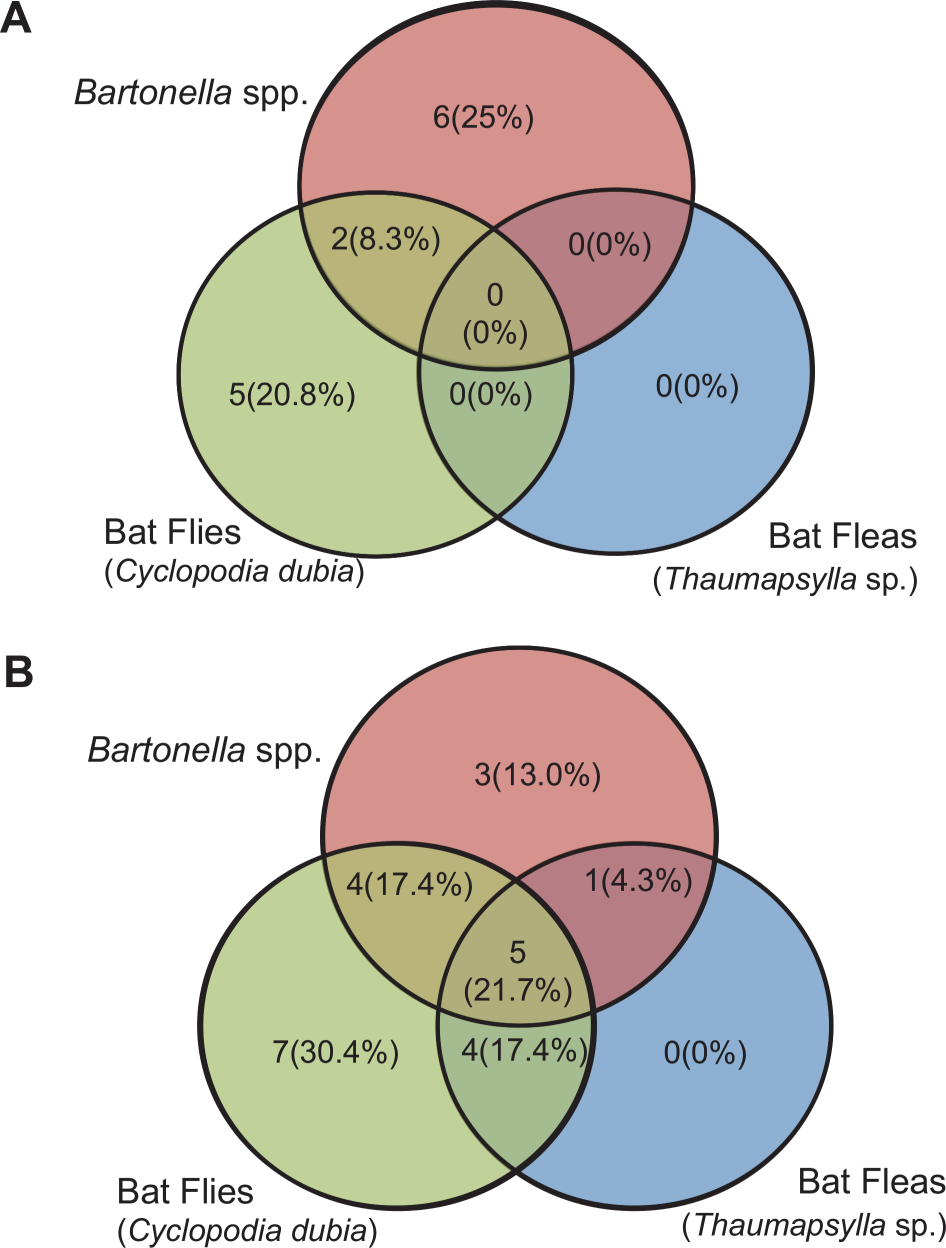
Venn-diagrams of infection/co-infection with bat flies, bat fleas, and *Bartonella* spp. across roosting sites for *Eidolon dupreanum*: (A) Angavobe, N = 24; (B) Angavokely, N = 24. Both raw numbers of infected individuals and prevalence (%) are indicated. Note that all sampled *P*. *rufus* from both Marovitsika and Ambakoana were negative for all infections (i.e. bat flies, bat fleas, and *Bartonella* spp.).

Blood samples from eight of 24 (33.3%) Angavobe *E*. *dupreanum* and thirteen of 23 Angavokely (56.5%) *E*. *dupreanum* were positive for *Bartonella* DNA by PCR confirmed with sequence for one or more genes (Table 1). *Bartonella* spp. prevalence did not vary significantly between Angavobe and Angavokely roosting sites as indicated by a chi-squared test for independence (X^2^ = 1.7029, df = 1, p-value = 0.1919) and Fisher’s exact test (p-value = 0.1468). In Angavobe, bats demonstrated both singular infections with *Bartonella* spp. and with bat flies, as well as simultaneous co-infection with bat flies and *Bartonella* spp. (Fig. 2). In Angavokely, *E*. *dupreanum* individuals hosted every possible combination of bat fly/flea/*Bartonella* spp. infection and co-infection save for singular flea infestations in the absence of other pathogens (Fig. 2). It should be noted that, prior to processing, bats were housed together with others from the same sample site in wooden transport cages, and ectoparasite sharing among individuals was easily facilitated.

No ectoparasites were recovered from either the 12 *Pteropus rufus* examined at the Marovitsika site or the 17 *P*. *rufus* sampled at the Ambakoana site (Table 1). As with ectoparasites, none of the 29 *P*. *rufus* samples (12 from Marovitisika, 17 from Ambakoana) were positive for *Bartonella* spp. by either molecular target (Table 1).

**Fig 3:**
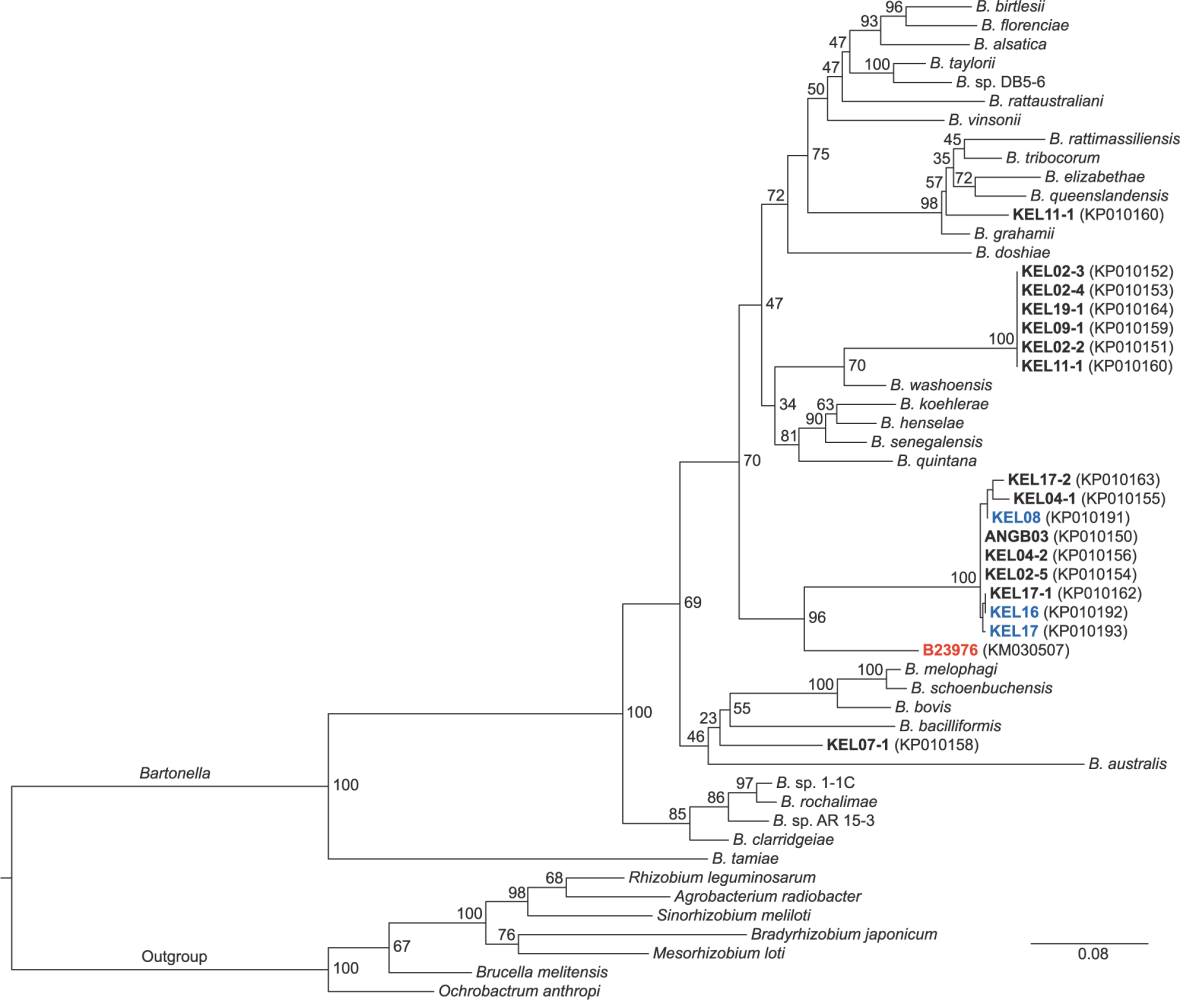
Maximum likelihood phylogeny of representative *gltA* genes of Rhizobiales (ingroup: *Bartonella* spp.) (RAxML, GTR+G model, partitioned by codon position) (*12*). KEL & ANGB—Madagascar samples. Blue: ex. *Eidolon dupreanum* (bat), Black: ex. *Cyclopodia dubia* (bat fly). RED comparative sequence ex. *Cyclopodia dubia* (bat fly) (*9*).

All *Bartonella* spp. sequences from *E*. *dupreanum* bats and associated *C*. *dubia* bat flies nested within or close to known major *Bartonella* lineages (Fig. 3) [17]. Although sequence data retrieved are insufficient to reach final *Bartonella* species identification, novel genotypes are present. Sequences (*gltA*) from sampled bats group with those retrieved from *Cyclopodia* bat flies.

## Discussion

The recent identification of bats as reservoirs for human pathogenic *Bartonella mayotimonensis* [9] validates further investigation of the zoonotic potential of *Bartonella* spp. in Chiropteran reservoirs. In Madagascar, insectivorous bats are known to roost in human residences, and both *P*. *rufus* and *E*. *dupreanum* are widely consumed as bushmeat, highlighting the extent of human-wildlife interface in the region [13].

In keeping with trends of persistent bacterial infection exhibited by bat-borne *Bartonella* elsewhere [3–5], we report high *Bartonella* spp. prevalence (57.4%) in a long-lived, cave-roosting *E*. *dupreanum* host (lifespan 10–20 years [13]). We correspondingly report no *Bartonella* infections in sympatric *P*. *rufus*, though our current sample size is too small to determine whether this absence is universal across the Madagascar population. Additionally, further study is needed to address whether these *Bartonella* spp. prevalence patterns are an artifact of phylogeny or ecology. The ability of *Eidolon* bats to serve as hosts for *Bartonella* spp. has now been documented in both tree-roosting [3] and cave-roosting environments—consistently in association with bat flies. *P*. *rufus* does not seem to host bat flies in Madagascar [18], although sampling has not been exhaustive enough to consider this absence a certainty. Tree-roosting *Pteropus* spp. are known to host bat flies throughout southeast Asia [19] and Australia [20], and investigation of *Bartonella* spp. infections in these populations will help address the relative influence of host genetic predisposition for *Bartonella* infection versus vector ecology.

In addition to pathogen prevalence in the host, we report *Bartonella* spp. infection in bat flies (*Cyclopodia dubia*) of *E*. *dupreanum*, simultaneous with *Bartonella* DNA absence in flea ectoparasites (*Thaumapsylla* sp.) of those same bats. Fleas are the confirmed vector for *Bartonella henselae*, the causative agent in cat scratch fever [21], and fleas of bats have been previously reported in association with *Bartonella* DNA [9,22]. In our study, both bat flies and fleas were host-specific and likely consumed host blood, although only flies tested positive for *Bartonella* spp., suggesting that the mechanisms by which arthropods host and transmit pathogens vary and impact their functionality as vectors. Sampling of flea ectoparasites was not extensive enough to assess the true extent of their ability, or lack of ability, to transmit *Bartonella* spp., and further experimental studies of the vector potential of both bat flies and fleas for *Bartonella* is warranted.

Finally, observed differences in the frequency of ectoparasite burden between sample sites for *E*. *dupreanum* indicated significantly higher rates of ectoparasite infection with both flies and fleas in Angavokely vs. Angavobe. These differences could result from ecological variation in both host density and/or climate between the two cave roosts. More extensive spatial sampling, in conjunction with climactic monitoring, in other *E*. *dupreanum* roost sites of varying size, temperature, and humidity across Madagascar will help elucidate habitat thresholds for ectoparasite invasion. In particular, sampling of *E*. *dupreanum* in reported tree roosts in central Madagascar will shed light on the extent to which roosting behavior limits bats’ abilities to support ectoparasites in this system [13]. If *Bartonella* spp. are, indeed, transmitted by bat fly vectors, such findings will have important implications for our understanding of the distribution, prevalence, and transmission dynamics of a potentially zoonotic pathogen.
